# Sex as Self-Injury Among Youth Clinic Visitors in Sweden

**DOI:** 10.1007/s10508-025-03325-w

**Published:** 2026-01-04

**Authors:** Ellen Ek, Cecilia Fredlund, Sofia Hammarström

**Affiliations:** 1 Medical School Graduate, Faculty of Medicine, Sahlgrenska Academy, University of Gothenburg, SE-413 90, Gothenburg, Sweden; 2 Department of Psychiatry in Linköping, and Department of Biomedical and Clinical Sciences, Linköping University, Linköping, Sweden; 3 Region Västra Götaland, Knowledge Centre for Sexual Health, Gothenburg, Sweden; 4 Faculty of Health and Society, Centre for Sexology and Sexuality Studies, Malmö University, Malmö, Sweden

**Keywords:** Sex as self-injury, Nonsuicidal self-injury, Adolescents, Sexual violence, Sexual and reproductive health, DSM-5

## Abstract

Earlier research has found that sex could be used as a means of self-injury with functions comparable to other self-injurious behaviors such as cutting or burning the skin. A suggested definition for sex as self-injury (SASI) is “a pattern of seeking sexual situations involving psychological or physical harm to themselves.” The research concerning SASI is sparse and the behavior is still in need of further understanding. The aim of this study was to investigate the prevalence of SASI among visitors to youth clinics in Sweden and the associations with sexual risk-taking, drug and alcohol consumption, and experience of violence. The study was based on data collected through the SEXual health Identification Tool (SEXIT) at youth clinics in multiple Swedish cities. A total of 813 participants were included in the study (Median = 19 years, SD 2.52 years, 734 identified as women, 78 as men, and 1 as other). Overall, 12.30% of the participants reported experience of SASI. SASI was most strongly associated with experience of sexual harassment or assault (OR 6.22), ever being diagnosed with a sexually transmitted infection (OR 3.07), drug use (OR 3.02), and experience of physical and/or psychological violence (OR 2.78). In conclusion, SASI is occurring among youth clinic visitors and is associated with factors that endanger young people’s physical and psychological well-being. Healthcare professionals need to have knowledge of SASI to offer proper help and support.

**Supplementary Information:**

The online version contains supplementary material available at 10.1007/s10508-025-03325-w.

## Introduction

Self-injurious behaviors are referred to in the literature by various terms, such as self-harm, deliberate self-harm, and nonsuicidal self-injury (NSSI). According to a recent systematic review and meta-analysis, NSSI is the most common term used, and the study reported an overall average prevalence for NSSI of 16% (Farkas et al., 2024). The reported prevalence was higher among females and in Asian populations, while it was lower in studies using nonvalidated questionnaires compared to those using questionnaires validated for self-injurious behaviors. This suggests that the definition used, as well as the studied population, has an impact on prevalence estimates. Additionally, a study of Swedish high school students suggests that the prevalence of self-injurious behaviors may have increased during the COVID-19 pandemic (Zetterqvist et al., 2021). Further research is necessary to understand NSSI and those engaging in it.

This need for further research was also stated in the fifth edition of the American Psychiatric Association’s (2013) *Diagnostic and Statistical Manual of Mental Disorders* (DSM-5), where NSSI disorder was proposed as its own diagnosis. However, there is ongoing discussion of whether the suggested definition of NSSI disorder is too narrow, thereby risking exclusion of behaviors with the same motive (Mann et al., 2022; Zetterqvist, 2015). One theory is that behaviors such as self-injury, risky sexual behaviors, substance use, aggression, binge eating, gambling, and impulsive spending share common etiologies (Bresin, 2020). These dysregulated behaviors have in common that they “prioritize short-term rewards over serious long-term consequences.” It has also been suggested that repeated sexual behaviors with associated physical and/or psychological pain and discomfort could fill the same purpose of emotional regulation as seen in NSSI, such as managing anxiety or other negative feelings (Jonsson et al., 2019). Such behavior could also be called sex as self-injury (SASI).

### Nonsuicidal Self-Injury

The first proposed criterion for the NSSI diagnosis suggested in DSM-5 states that the individual has engaged in intentional self-inflicted damage that has caused injury to the body that is likely to induce pain, bleeding, or bruising (American Psychiatric Association, 2013). This behavior should have occurred on at least five days in the last year. The nonsuicidal intent of the behavior should either be stated or can be inferred from the person having engaged in the behavior previously and thereby learnt that it is not likely to result in death. Other criteria involve the expectation of obtaining relief from negative feelings, resolving interpersonal difficulties, or inducing positive feelings; the action not being socially sanctioned; and the behavior or its consequences causing clinically significant distress.

Another categorization of self-injury used in the literature is direct or indirect self-injury. Direct self-injury usually refers to behaviors directed to the surface of the body, like cutting or burning the skin, hitting, banging one’s head, or pulling one’s hair, while indirect self-injury is defined as behaviors that are damaging and unhealthy in the long term while not necessarily causing immediate damage to body tissue (D’Agostino et al., 2020; St Germain & Hooley, 2012). Examples of indirect self-injury include eating disorders, involvement in abusive relationships, over-exercise, and the use of drugs or alcohol. One suggestion is to consider injury via sexual activity, as well as other forms of harm inflicted by “another,” such as injury from an animal or a physical fight, under the term “NSSI by proxy” (Mann et al., 2022). NSSI by proxy has been described as deliberate destruction of one’s own body tissue through the actions of someone else, human or animal, without suicidal intentions and for reasons not socially sanctioned. NSSI has been shown to be a significant risk factor for both suicide and suicide attempts (Hawton et al., 2015; Klonsky et al., 2013; Zetterqvist, 2015).

### Sex as Self-Injury

A suggested definition of SASI is “when a person has a pattern of seeking sexual situations involving psychological or physical harm to themselves. The behavior causes significant distress or impairment in school, work, or other important areas” (Fredlund et al., 2017). The study, in which this definition was presented, included Swedish adolescents (mean age 18 years) attending their third year of high school in 2014. It found that 3.2% of the girls and 0.8% of the boys reported having used sex to intentionally harm themselves on at least one occasion. In a small pilot study including 50 American college students, 12% reported sex as self-injury (Mellin & Young, 2024).

Manifestations of SASI have been described in qualitative research as including both psychological and physical harm (Fredlund et al., 2020). Psychological harm could manifest as sexual contact without an inner feeling of wanting, desire, or attraction to the other person, while physical harm could include physical violence in sexual situations such as hitting, spitting, burning, or strangling, as well as unprotected sex. Some individuals described SASI as “self-elected rape” where the person themself could seek out violent sexual encounters or situations similar to earlier experiences of rape, with the motives of emotional regulation or positive or negative confirmation (Fredlund et al., 2020). The most common underlying motives described for SASI involve emotional regulation, such as to stop bad feelings, relieving feelings of being numb or empty, being able to feel something (even if it is pain), and feeling in control (Jonsson et al., 2019); these are similar to the motives seen in NSSI. A meta-analysis studying the prevalence of different functions of NSSI found emotional regulation to be one of the most common motives for NSSI (Taylor et al., 2018). Pleasure has not been stated as a motive for SASI, thus distinguishing it from other described types of sexual behavior (Fredlund et al., 2020). Social motives such as getting attention or getting a reaction from someone were more commonly reported by individuals engaged in SASI than by those engaged in NSSI (Jonsson et al., 2019).

SASI has been associated with earlier experiences of sexual abuse as well as sexual risk-taking such as a higher number of sexual partners (Fredlund et al., 2017; Zetterqvist et al., 2018). When adolescents reporting SASI were compared to those reporting NSSI, adolescents in the SASI group more commonly reported experiences of penetrative sexual abuse and had higher levels of self-reported trauma symptoms (Zetterqvist et al., 2018). SASI might also increase the risk of further sexual violence and revictimization (Fredlund et al., 2020), which highlights a need for early recognition of these adolescents to enable both remedying and preventive care.

In conclusion, few studies exist that investigate the prevalence of SASI and there is currently no knowledge of how common it is in clinical populations. It can be assumed that youth clinics are places where people with SASI might seek care, thus places that should be able to offer help and support. At present, however, it is not known how prevalent SASI is or what risk factors are associated with SASI in this population.

### Present Study

This study aimed to investigate the prevalence of SASI among visitors to Swedish youth clinics, as well as to examine potential risk factors of SASI. The research question was: What is the prevalence of SASI among visitors in Swedish youth clinics and how is it associated with sexual risk-taking, experience of violence, as well as alcohol and drug use?

## Method

This is a survey-based study that used the SEXual Health Identification Tool (SEXIT) collected from youth clinics in the Swedish regions of Gothenburg and Värmland. In Sweden, there are approximately 250 youth clinics offering youths aged 13–25 advice on contraceptives, free condoms, tests for sexually transmitted infections (STIs) or pregnancy, medical advice, and counseling. Healthcare professionals (HCPs) in youth clinics mainly include midwives, nurses, and counselors but doctors, psychologists, and other professionals may also be available. Historically, most youth clinic visitors have been women (89%), often explained by a focus on contraceptive services (Wendt & Leijen, 2015).

### Participants

The study included visitors to 15 Swedish youth clinics, 14 of them in the region of Värmland and one in central Gothenburg. These clinics were located in communities of various sizes: the second largest city in Sweden (Gothenburg, approximately 608,000 inhabitants), a medium-sized town (Karlstad, approximately 67,000 inhabitants), and smaller communities in Värmland. A total of 991 questionnaires were collected, comprising 458 from Värmland and 533 from Gothenburg. However, 132 individuals were excluded from the study since they had not correctly answered the index question concerning SASI. An incorrect answer was defined as not answering at all, ticking more than one box, or writing on the questionnaire outside of the boxes. Most of these individuals, 123 in total, had not yet had their sexual debut and, as a result of this, did not answer any questions in the second half of the SEXIT questionnaire. A further 46 individuals were excluded as they had answered “Don’t know” to the question on SASI. In total, 813 participants were included in the study. The participants' age, gender, sexual orientation, and living situation are given in Table 1. Mean age was 19.44 years (SD 2.31) among participants reporting SASI and 18.92 years (SD 2.54) among the reference group. A total of 734 participants identified as female, 78 as male, and 1 as other. For exact phrasing of the questions and answers, please see the SEXIT questionnaire in its entirety in the Appendix.

**Table 1:** Background characteristics of adolescents with experience of sex as self-injury (SASI) and not (non-SASI)

	SASI		Non-SASI	
	n	%	n	%
*Age in years*				
Under 18	25	25.25	224	31.55
18–21	55	55.56	355	50.00
22–25	19	19.19	131	18.45
Mean age in years	19.44		18.92	
Median age in years	20		19	
*Sex*				
Female	93	93.94	641	89.90
Male	6	6.06	72	10.10
Sexual orientation				
Heterosexual	78	80.41	630	90.65
Non-heterosexual	19	19.59	65	9.35
*Living situation*				
With parent/guardian	45	48.39	434	62.72
With friend/partner	18	19.35	107	15.46
Alone	30	32.26	151	21.82

### Procedure

The data from Värmland were collected in late 2019, and the data from Gothenburg were collected between late 2021 and early 2022. This study was based on SEXIT, a self-report questionnaire which is used as a conversational tool at the youth clinics included in the study. The use of SEXIT is part of the regular routines at the youth clinics, and the questionnaires were issued as part of their development work. Participants were asked to fill out the SEXIT questionnaire during their visit to the clinic, after which an HCP went through the answers together with the participant, asked follow-up questions, and noted all relevant information in the patient records. Filling out the questionnaire was voluntary and was offered to all new visitors regardless of the reason for their visit. The participants were informed of the procedure, before filling out the questionnaire. For this study, the response rate was not recorded, as the data were not initially collected to be used in research; however, a previous study using SEXIT reported a response rate of 86% in youth clinics (Hammarström et al., 2019). Before the data were analyzed, a power analysis was performed indicating that the sample size was sufficient for answering the question on the prevalence of SASI in this group.

### Measures

The first version of SEXIT was developed during 2016 by the Knowledge Centre for Sexual Health in Region Västra Götaland. Its purpose is to serve as a tool for HCPs, giving them a structured and standardized way to ask questions about sexual health, risk-taking, and violence. In addition to the questionnaire, SEXIT includes two other components: training of HCPs before the implementation of the tool, and a handbook to support them in using SEXIT. The assessment tool has been validated and evaluated in the youth clinic setting through discussions by an expert panel and by focus groups of youths (Hammarström, et al., 2019, 2021, 2022a, 2022b). Further testing and development resulted in the creation of SEXIT 3.0, which includes a question on SASI. However, SEXIT 3.0 has not yet been validated.

SEXIT 3.0 contains a total of 22 questions concerning sociodemographic factors, alcohol and drug use, experiences of violence, and sexual health. The index question for this study was “Have you used sex to harm yourself intentionally or to deal with difficult emotions? Also applies for sex via phone or computer.” The full English translation of the SEXIT 3.0 questionnaire is given in the Appendix.

### Statistical Analysis

Explorative bivariate analyses were initially conducted comparing the group with experience of SASI to the control group, regarding each of the questions in the questionnaire separately. Descriptive statistics and odds ratios (ORs) comparing the SASI group with the reference group are presented in Tables 2–3. Eight variables (drug use, exposure to controlling behavior, experience of psychological and/or physical violence, experience of sexual harassment and/or assault, violence in the family, early sexual debut, high number of sexual partners, and occurrence of STI) were found to be most significantly related to SASI (*p *< 0.001) in the univariate analyses and were further examined using multivariate logistic regression to identify potential risk factors for SASI. As SEXIT is developed to identify sexual risk-taking, most of these variables were expected to have some association with SASI. The cutoff *p* < 0.001 was chosen to focus on those variables which were most strongly associated with SASI specifically, and not with sexual risk-taking in general. These results can be found in Table 4.

**Table 2:** Alcohol, drug use and experiences of violence among adolescents with sex as self-injury (SASI) and not (non-SASI)

	SASI		Non-SASI		OR	95% CI
	n	%	n	%		
*Alcohol use in the last year*						
Less than twice a week	86	86.87	651	93.00	–	–
More than twice a week	13	13.13	49	7.00	2.01^*^	1.05–3.85
Drug use	43	43.00	96	13.54	4.82^***^	3.07–7.56
Being controlled	29	31.52	60	8.67	4.85^***^	2.90–8.10
Controlling others	2	2.15	11	1.56	1.38	0.30–6.34
Violence (physical/psychological)	66	66.00	178	24.96	5.83^***^	3.73–9.12
Violence against others	9	9.78	12	1.72	6.19^***^	2.53–15.13
Sexual harassment and/or assault	87	88.78	318	44.73	9.77^***^	5.13–18.62
Violence within family	28	30.11	86	12.74	2.95^***^	1.79–4.85

^*^p < 0.05, ***p < 0.001

**Table 3:** Sexual risk-taking and ill health among adolescents with sex as self-injury (SASI) and not (non-SASI)

	SASI		Non-SASI		OR	95% CI
	n	%	n	%		
Sexual debut < age 15	36	36.36	144	20.40	2.23^***^	1.42–3.49
More than two sexual partners in the last year	53	54.64	200	28.45	3.03^***^	1.97–4.67
*Use of protection against* *STI*						
Always	15	15.46	163	23.49	–	–
Sometimes	33	34.02	275	39.63	1.30	0.69–2.47
Never	49	50.52	256	36.89	2.08^*^	1.13–3.83
*Use of protection against* *Pregnancy*						
Always	68	73.12	514	79.32	–	–
Sometimes	20	21.51	104	16.05	1.45	0.85–2.50
Never	5	5.38	30	4.63	1.26	0.47–3.36
STI diagnosis	34	34.34	59	8.70	5.49^***^	3.35–8.99
Unplanned pregnancy	10	10.31	24	3.42	3.24^**^	1.50–7.01
Received payment for sex	7	7.29	3	0.42	18.56^***^	4.72–73.07

^*^p < 0.05, ^**^p < 0.01, ^***^p < 0.001. STI = sexually transmitted infection

**Table 4:** Risk factors of sex as self-injury in multivariate logistic regression including odds ratio (OR)

	OR	95% CI
Drug use	3.02^***^	1.67–5.45
Being controlled	2.45^*^	1.24–4.85
Violence (physical/psychological)	2.78^***^	1.55–4.98
Sexual harassment and/or assault	6.22^***^	3.04–12.73
Violence within family	1.15	0.60–2.22
Sexual debut before age 15	0.80	0.43–1.50
More than two sexual partners in the last year	1.97^*^	1.14–3.41
Sexual transmitted infection	3.07^***^	1.64–5.77
R2, Cox & Snell	0.19	
R2, Nagelkerke	0.36	

^*^p < 0.05, ^**^p < 0.01, ^***^p < 0.001

A total of 813 participants were included in the analyses, but there was an internal loss on certain questions as participants had answered “Don’t know” or failed to answer some questions entirely. The largest internal loss occurred on question 11B, regarding sexual assault, where 6.5% answered “Don’t know” or gave no answer at all. The questions on exposing someone else to sexual harassment or assault were also excluded from the analysis due to small numbers. It should be noted that those who reported experience of physical or psychological violence, or both, were merged into one group. Version 28 of IBM SPSS was used for statistical analysis.

## Results

The prevalence of participants stating that they had used sex to intentionally harm themselves or to deal with difficult emotions on any occasion was 12.30% overall (*n *= 100), 12.67% among women, and 7.69% among men.

As seen in Table 2, participants in the SASI group were more likely to report drinking alcohol twice a week or more (OR 2.01). There was a difference between the SASI and the reference group regarding reported drug use, with participants in the SASI group more commonly reporting ever having used drugs (OR 4.82).

Participants with SASI had been more exposed to controlling behavior (OR 4.85) and were more likely to have experienced some kind of violence, either physical and/or psychological (OR 5.83). Exposing someone else to violence was also more common in the SASI group (OR 6.19).

Participants in the SASI group reported more experience of sexual harassment and/or assault (OR 9.77). Moreover, 30% of the participants in this group reported that during childhood or adolescence someone in their family had been exposed to any form of violence, which was more common than in the reference group (OR 2.95).

As seen in Table 3, participants in the SASI group were more likely to have had their sexual debut before the age of 15 years (OR 2.23). The mean age for the first sexual encounter was 15.02 years (SD 1.60 years) in the SASI group and 15.97 years (SD 1.79 years) in the reference group. SASI was associated with having a higher number of sexual partners in the last year (OR 3.03, for more than 2 partners). The mean number of partners in the last year was 4.22 (SD 3.87) in the SASI group and 2.20 (SD 2.19) in the reference group.

Regarding use of protection against STI (use of condoms or dental dams), participants in the SASI group were more likely than the non-SASI group to never use protection (OR 2.08). No significant differences were seen regarding protection against pregnancy, but SASI was associated with experience of STI (OR 5.49) as well as unplanned pregnancy (OR 3.24). Experience of sex for compensation was more common among participants in the SASI group (OR 18.56), though only 10 individuals in total reported sex for compensation.

Eight variables were included in the logistic regression as potential risk factors for SASI: drug use, exposure to controlling behavior, experience of psychological and/or physical violence, sexual harassment and/or assault, violence in the family, sexual debut before the age of 15, more than two sexual partners in the last year, and occurrence of STI. The strongest associated factors for SASI were experience of sexual harassment and/or assault (OR 6.22), followed by STI diagnosis (OR 3.07), drug use (OR 3.02), and experience of physical and/or psychological violence (OR 2.78). Experience of being controlled (OR 2.45) and having more than two sexual partners in the last year (OR 1.97) were also found to be significantly associated with SASI in the model. Violence within the family and early sexual debut did not show a significant association with SASI when examined collectively with the other six factors. These results are shown in Table 4.

## Discussion

The aim of this study was to estimate the self-reported prevalence of sex as self-injury among youth clinic visitors in Sweden and to examine potential risk factors of SASI, such as abuse, drug and alcohol consumption, and sexual risk-taking.

The prevalence of SASI was found to be 12.30% among the visitors of youth clinics. This is higher than previous findings among Swedish high school students (Fredlund et al., 2017), but in line with results found in a pilot study assessing the frequency of SASI among US college students (Mellin & Young, 2024). It seems reasonable to assume that the frequency of SASI among visitors to youth clinics is likely to be higher than among high school students since the population is actively seeking health care for reasons often related to their sexual or psychological health (Wendt & Leijen, 2015), which could be connected to SASI. Previous studies have found that individuals visiting youth clinics comprise a vulnerable group regarding experiences of violence and sexual ill health (Hammarström et al., 2019, 2022a, 2022b). The phrasing of the question can also have an impact on the observed prevalence. For example, questionnaires with checklists of different forms of NSSI will record a higher prevalence of the behavior than simply asking about NSSI in a single question (Robinson & Wilson, 2020). The fact that the questionnaire was part of a clinical setting, with HCPs wanting to discuss the answers with the youths, might also have affected their answers. Social desirability bias might have affected the responses given by participants, and a desire to limit discussion of uncomfortable topics could have compelled them to not answer truthfully. The effect this has on the results can, unfortunately, only be guessed.

Gender was not assessed as a risk factor for SASI in this study, as only a very small part of the study population were men (9.59%). This is related to the fact that most visitors to youth clinics are women (Wagenius et al., 2019; Wendt & Leijen, 2015), and most of our study population were women. However, it is worth noting that 93 out of 100 participants who reported SASI in this study were women. Results seen in earlier research also report SASI to be more common among women (Fredlund et al., 2017). This suggests that youth clinics might be a good way to reach out to young women with risk factors of SASI; but, conversely, young men in need of help and support might be excluded. This could be remedied by screening for SASI in school health care as a way of also reaching men and boys. Studies investigating this approach are currently underway. Another approach is health clinics established exclusively for young men, with the potential to attract men more effectively. Previous research has shown that some men prefer clinics targeted toward men only, while others prefer clinics for men and women, but also that the men emphasize the importance of a respectful encounter with HCPs when seeking care (Buzi & Smith, 2014). Another aspect regarding gender that was not assessed in this study was the occurrence of SASI among transgender youth. Analyzing the association of transgender identity and SASI was not possible since the SEXIT questionnaire does not ask participants to specify whether they identify as cis- or transgender.

The present study found the experience of sexual harassment and/or assault as well as the experience of physical and/or psychological violence to be significant risk factors for SASI. This confirms previous research indicating that SASI is associated with the experience of violence, especially earlier experiences of sexual abuse (Fredlund et al., 2017, 2020; Jonsson et al., 2019). A study published in 2023 found that the connection between SASI and sexual violence can be perceived as a loop: SASI can escalate into sexual violence, and earlier experiences of sexual violence can lead to SASI because of a shift in boundaries or a way of regaining control over one’s body and sexuality after abuse (Hedén et al., 2023).

The present study also found SASI to be associated with the use of physical and/or psychological violence against others. Previous studies have suggested a “cycle of maltreatment” which indicates that maltreatment in childhood increases the risk of perpetuating violence (Augustyn et al., 2019; Madigan et al., 2019; Thornberry et al., 2013). This has, however, mainly been studied regarding parents and their children, where the mechanisms of transmission and possible moderators are not yet fully understood.

SASI was significantly associated with lower age at sexual debut, a higher number of sexual partners, lower use of protection against STI (use of condoms or dental dams), occurrence of STI, and unplanned pregnancy. These findings are in line with results from previous studies and show that sexual risk-taking is common and related to SASI (Fredlund et al., 2017, 2020; Jonsson et al., 2019). This suggests a particular need for preventive measures in this group, and patients should urgently be offered contraceptive counseling to avoid STI and unplanned pregnancies.

Finally, drug use was also found to be a significant risk factor for SASI. A recent study found that youths who report polysubstance use have a higher risk of experiencing negative alcohol-related sexual experiences, such as having unprotected sex, sexual activities they would not have done when sober, or regretting sexual activities (Lewis et al., 2023). Use of both stimulants and marijuana with alcohol resulted in a higher increase in risk than use of marijuana and alcohol, which in turn had a higher risk than use of alcohol alone.

Studies show that there are barriers and delays when seeking help after sexual abuse, where lack of resources or competence among HCPs has been stated as a hindrance to appropriate care (Rajan et al., 2021; Wright et al., 2022). Recent research has found that individuals with experience of SASI are met with this same deficiency in knowledge (Fredlund & Jonsson, 2023). These individuals report that they want to be asked about their sexual health in general, and about SASI in particular. It was also important for them to have a word to describe their behavior, to understand the function of the behavior, to be met with respect and empathy, and to get help with underlying causes of the behavior such as earlier experiences of traumatization. Similarly, women who disclose experiences of intimate partner abuse to HCPs have been found to need and expect kindness and empathy, non-judgmental behavior, practical support, and empowerment (Tarzia et al., 2020).

Awareness of SASI and the factors associated with it is important both for enabling further research on the topic and for the ability to offer good clinical practice. Just to get a word for SASI is helpful to leave the behavior but also necessary to get help and support, as has been described in earlier research (Fredlund et al., 2023). Self-injurious behaviors could take different forms and change over time; for one person with NSSI, the behavior could change to SASI, fulfilling the same need of emotional regulation. If this is not addressed in treatment, the person will not be helped (Fredlund et al., 2020). Further, a person might not always be aware of SASI as self-injurious behavior. It has been described that a person, afterward, for example in treatment, gets the understanding of why it was so hard to leave the behavior of self-destructive sexual relations (Fredlund et al., 2020). To get a word for SASI could then be helpful to see the patterns and know what is needed in treatment. HCPs need training concerning SASI to be able to offer a safe and knowledgeable space where vulnerable individuals may share their experiences. It is also necessary to have clinical routines that facilitate raising the subject and that enable identification of individuals with SASI, who often require particular attention. One such resource is the SEXIT toolkit (Hammarström et al., 2022b).

### Strengths and Limitations

This study is unique in its focus on investigating the prevalence of SASI in a sample of youth clinic visitors. Even though the study was not representative of the general population, one strength was the collection of answers from different parts of Sweden, including the second largest city in the country, a medium-sized town, and several smaller rural communities.

There are several limitations of this study. Firstly, version 3.0 of SEXIT has not yet been validated. The question on SASI was added to the questionnaire in 2019 and was not included in the previous versions. Secondly, to facilitate the analyses, all “Don’t know” answers were excluded. A total of 46 individuals answered the question on SASI with “Don’t know”, and several other questions were also given this answer. It would be interesting to know more about this group and what makes them unsure of their answers. For example, 74 individuals answered “don’t know” on one or several questions regarding violence (physical, psychological, or sexual). Any number of victims might also be hidden behind answers of “no” to these questions, as youths might not recognize violence or assault for what it actually is. Further research, preferably of a more qualitative nature, is needed to investigate this topic. Thirdly, the definition of sex used in the SEXIT questionnaire is broad, essentially including anything that the participant themselves considers as sex. By extension, this makes it difficult to know exactly what participants in the study refer to when they answer yes on the questions of sex as self-injury.

Finally, as the data contained many variables, multiple tests of significance were performed. This increases the risk of type I errors or false positives. As a way of limiting this risk, only eight variables were included in the multivariate logistic regression.

### Conclusions

In conclusion, this study found that 12.30% of visitors to youth clinics have experience of SASI and that these individuals are vulnerable when it comes to other parts of their physical, psychological, and sexual health. SASI was significantly associated with drug use, experience of controlling behavior, violence, sexual harassment and assault, as well as sexual risk-taking. This should be of interest to all HCPs, especially those working at youth clinics or in other settings where they encounter young people, since individuals with experience of SASI comprise a highly vulnerable group. These individuals need help and support, and HCPs need training and knowledge to provide this in a respectful and well-informed way.

## Supplementary Information

Below is the link to the electronic supplementary material.

Supplementary file1 (PDF 199 KB)

## Data Availability

Data available upon request. However, an ethical review may be necessary to adhere to the Swedish General Data Protection Regulation.
