# Supplementary material for: Sex as Self-Injury Among Youth Clinic Visitors in Sweden
Source: Arch Sex Behav. 2026 Jan 4;55(1):109–17. doi: 10.1007/s10508-025-03325-w (PMC12916961; doi:10.1007/s10508-025-03325-w)
Supplement: Supplementary file 1 — Supplementary file1 (PDF 199 KB) [file 10508_2025_3325_MOESM1_ESM.pdf]

# Interview about sexual health and experiences of violence

We are asking you these questions in order to give you better care and support.
You can choose which ones you want to answer and which answer fits you best.

| | | | | | |
|---|---|---|---|---|---|
| **1. How old are you?** | ________ (years) | | | | |
| **2. What is your gender?** By gender we mean gender identity – the gender you feel like you are. | Woman ☐ | Man ☐ | Other ☐ ______________ | | Don't know ☐ |
| **3. What is your sexual orientation?** | Heterosexual ☐ | Homosexual ☐ | Bisexual ☐ | Other ☐ | Don't know ☐ |
| **4. What is your living situation?** | Live with my parents/ guardian ☐ | Live with a friend/ partner ☐ | In a family home/ HVB (home for care or residence) ☐ | Alone ☐ | Other arrangement ☐ |
| **5. How frequently have you drunk alcohol in the past year?** | | Never ☐ | Once a month or less ☐ | 2–4 times a month ☐ | Twice a week or more ☐ |
| **6. Have you used drugs?** Cannabis, amphetamines, heroin, LSD, anabolic steroids, poppers or other drugs. | | | Never ☐ | Yes, more than a year ago ☐ | Yes, during the past year ☐ |

| | | | |
|---|---|---|---|
| **7. Has anyone restricted or controlled you in your choice of partner, company, clothes, or leisure activities, for example?** | Yes ☐ | No ☐ | Don't know ☐ |
| **8. Have you restricted or controlled someone as described above?** | Yes ☐ | No ☐ | Don't know ☐ |
| **9. Has anyone used physical or psychological violence against you?** Physical violence includes being beaten, kicked, pushed, or injured in some other way. Psychological violence includes being insulted, degraded, threatened, harassed, or bullied. Psychological violence can also be carried out via a phone or computer. **More than one answer possible.** | Yes, physical violence ☐ | No ☐ | Don't know ☐ |
| | Yes, psychological violence ☐ | No ☐ | Don't know ☐ |
| **10. Have you subjected someone to physical and/ or psychological violence as described above?** | Yes ☐ | No ☐ | Don't know ☐ |
| **11. Have you been subjected to any of the following against your will?** Includes via phone or computer. **- seen someone expose themselves, been groped by someone, received or sent intimate photos** | Yes ☐ | No ☐ | Don't know ☐ |
| **- masturbated someone or had vaginal, oral or anal sex** | Yes ☐ | No ☐ | Don't know ☐ |
| **12. Have you groped someone, sent them or demanded intimate photos from them, or carried out some other sexual act against their will?** | Yes ☐ | No ☐ | Don't know ☐ |
| **13. Did you during your childhood experience anyone in your family being subjected to psychological, physical or sexual violence?** | Yes ☐ | No ☐ | Don't know ☐ |

sexit

Having sex can mean having sexual intercourse vaginally, orally or anally. But sex can also be many other things, such as masturbating with someone, necking, caressing or being caressed. This can be when you meet in person but **also via a phone or computer**. You decide yourself when was the first time for you.

## If you have not had sex with anyone you have now completed the questionnaire.

| | | | | | |
|---|---|---|---|---|---|
| **14. How old were you when you first had sex with someone?** | | ________ (years) | | | |
| **15. How many people have you had sex with in the past 12 months?** Also applies for sex via phone or computer. | | ________ (number) | | | |
| **16. How often do you and your partners use a condom or dental dam as protection against sexually transmitted diseases?** | | | Always ☐ | Sometimes ☐ | Never ☐ |
| **17. How often do you and your partners use contraceptives?** | Always ☐ | Sometimes ☐ | Never ☐ | Don't know ☐ | Not applicable ☐ |
| **18. Do you have or have you had chlamydia, gonorrhoea, syphilis, hepatitis or HIV?** | | | Yes ☐ | No ☐ | Don't know ☐ |
| **19. Have you or has any partner of yours had an unplanned pregnancy?** | | | Yes ☐ | No ☐ | Don't know ☐ |
| **20. Have you used sex to harm yourself intentionally or to deal with difficult emotions?** Also applies for sex via phone or computer. | | | Yes ☐ | No ☐ | Don't know ☐ |
| **21. Have you received compensation or payment for sexual services?** Also applies for sex via phone or computer. Compensation can be money, alcohol, cigarettes, drugs, lodging, food, things, travel etc. | | | Yes ☐ | No ☐ | Don't know ☐ |
| **22. Have you given compensation or paid for sexual services in Sweden or abroad?** Also applies for sex via phone or computer. | | | Yes ☐ | No ☐ | Don't know ☐ |

## You are welcome to write down any of your own thoughts that you would like us to talk about:

______________________________

______________________________

______________________________

**Notes by staff (Personalens anteckningar)**
(flera svar möjliga)

| A | B | C | D | E | F | G | H | Bokat besök | Drop-in/ obokat besök |
|---|---|---|---|---|---|---|---|---|---|
| ☐ | ☐ | ☐ | ☐ | ☐ | ☐ | ☐ | ☐ | ☐ | ☐ |

*SEXIT Version 3.0 developed by the Knowledge Centre for Sexual Health, Region Västra Götaland.*

View publication stats
